# Whole genome sequence analysis indicates recent diversification of mammal-associated *Campylobacter fetus* and implicates a genetic factor associated with H_2_S production

**DOI:** 10.1186/s12864-016-3058-7

**Published:** 2016-09-06

**Authors:** Linda van der Graaf–van Bloois, Birgitta Duim, William G. Miller, Ken J. Forbes, Jaap A. Wagenaar, Aldert Zomer

**Affiliations:** 1 Department of Infectious Diseases and Immunology, Faculty of Veterinary Medicine, Utrecht University, Utrecht, The Netherlands; 2 WHO Collaborating Centre for Campylobacter/OIE Reference Laboratory for Campylobacteriosis, Utrecht, The Netherlands; 3 U.S. Department of Agriculture, Produce Safety and Microbiology Research Unit, Agricultural Research Service, Albany, CA USA; 4 School of Medicine and Dentistry, University of Aberdeen, Aberdeen, UK; 5 Central Veterinary Institute of Wageningen UR, Lelystad, The Netherlands

**Keywords:** *Campylobacter fetus*, Bovine Genital Campylobacteriosis, Subspecies differentiation, Core genome SNP analysis, H_2_S production, Cysteine transporter

## Abstract

**Background:**

*Campylobacter fetus* (*C. fetus*) can cause disease in both humans and animals. *C. fetus* has been divided into three subspecies: *C. fetus* subsp. *fetus* (Cff), *C. fetus* subsp. *venerealis* (Cfv) and *C. fetus* subsp. *testudinum* (Cft). Subspecies identification of mammal-associated *C. fetus* strains is crucial in the control of Bovine Genital Campylobacteriosis (BGC), a syndrome associated with Cfv. The prescribed methods for subspecies identification of the Cff and Cfv isolates are: tolerance to 1 % glycine and H_2_S production.

**Results:**

In this study, we observed the deletion of a putative cysteine transporter in the Cfv strains, which are not able to produce H_2_S from L-cysteine. Phylogenetic reconstruction of the core genome single nucleotide polymorphisms (SNPs) within Cff and Cfv strains divided these strains into five different clades and showed that the Cfv clade and a Cff clade evolved from a single Cff ancestor.

**Conclusions:**

Multiple *C. fetus* clades were observed, which were not consistent with the biochemical differentiation of the strains. This suggests the need for a closer evaluation of the current *C. fetus* subspecies differentiation, considering that the phenotypic differentiation is still applied in BGC control programs.

**Electronic supplementary material:**

The online version of this article (doi:10.1186/s12864-016-3058-7) contains supplementary material, which is available to authorized users.

## Background

The pathogen *Campylobacter fetus* (*C. fetus*) can cause disease in both animals and humans. In humans, *C. fetus* infections vary from acute diarrhea to systemic illness [1]. In animals, *C. fetus* infections can cause abortion and infertility, mainly in cattle and in sheep [2]. The mammal-associated *C. fetus* subspecies are *C. fetus* subsp. *fetus* (Cff) and *C. fetus* subsp. *venerealis* (Cfv) [3], whereas *C. fetus* subsp. *testudinum* (Cft) is associated with reptiles [4]. *C. fetus* subsp. *venerealis* includes a biochemical variant, designated *C. fetus* subsp. *venerealis* biovar intermedius (Cfvi) [5].

*C. fetus* subsp. *venerealis* has been described to be the causative agent of Bovine Genital Campylobacteriosis (BGC), associated with infertility and abortion in cattle [6]. BGC is notifiable to the World Organisation for Animal Health (OIE). A crucial element in the BGC control program relies on the subspecies identification of *C. fetus* isolates. Currently, the methods prescribed by the OIE to differentiate Cff, Cfv and Cfvi are tolerance to 1 % glycine and H_2_S production [7]: Cff is tolerant to 1 % glycine and able to produce H_2_S, Cfv is not tolerant to 1 % glycine and not able to produce H_2_S and Cfvi is not tolerant to 1 % glycine (like Cfv) and able to produce H_2_S (like Cff). The biochemical tests are hampered by poor reproducibility [8], and the phenotypes are not completely consistent with the genomic characteristics of the *C. fetus* strains, since phenotypically-identified Cff strains were genotypically identical with Cfv strains [9]. An obvious distinguishing and important feature of *C. fetus* cells is the surface layer (S-layer), which is considered to be associated with the pathogenicity of *C. fetus* strains [10]. *C. fetus* cells can express two types of surface array proteins (Sap), which correlates with the serotypes of the bacterium; Cfv strains are serotype A; Cff strains may be either serotype A or serotype B and rarely serotype AB; and Cft strains are serotype A, serotype B or serotype AB [11, 12]. The molecular method multi-locus sequence typing (MLST) was recommended to differentiate Cff and Cfv strains [8]. However, a recent study showed that the current MLST scheme was not able to reliably differentiate the *C. fetus* subspecies, as a Cff strain was isolated with the Cfv-associated MLST ST-4 genotype [13].

Whole-genome analysis provides fine-scale resolution of bacterial genomes and allows the calculation of evolutionary events, as shown for *C. coli* and *C. jejuni* [14]. Whole-genome analysis has improved, and will continue to improve our understanding of the features that distinguish *C. fetus* subspecies and the evolutionary forces that have acted on *C. fetus* over time. In this study, we performed a core genome single-nucleotide polymorphisms (SNPs) analysis of 42 *C. fetus* Cff and Cfv genomes to identify subspecies-specific SNPs. We performed a SNP-based phylogenetic analysis of the core genomes and a BEAST analysis to estimate the divergence dates of Cff and Cfv strains. Additionally, we investigated whether the genomes contain specific SNPs or genes that could be associated with the biochemical tests and different clinical features of the *C. fetus* strains.

## Methods

### Bacterial strains and whole genome sequencing

In this study, 42 *C. fetus* strains from different countries and sources were included (Table 1). The strains were biochemically characterized (except the NCBI GenBank strains H1-UY, 642–21, B6, and 99/541), using H_2_S production in medium amended with 0.02 % cysteine-HCl and 1 % glycine tolerance, as described before [15]. Genotypic subspecies characterisation was performed using MLST [8] and AFLP [16]. The whole genome sequence data of all *C. fetus* genomes was published previously [9, 12, 17]. Briefly, the *C. fetus* genomes (except the genomes with accession numbers starting with ERR and the genomes downloaded from NCBI GenBank) were sequenced using a Roche 454 GS-FLX+ Genome sequencer with Titanium chemistry and assembled into contigs using the Newbler Assembler (version 2.6). The genomes with accession numbers starting with ERR were sequenced using an Illumina Hiseq and assembled into contigs with the Velvet assembler in an established pipeline at the Sanger Institute. The genomes of four *C. fetus* strains (04/554, 97/608, 03/293 and 01/165) were closed by using a PacBio RS sequencer and assembled into contigs using Quiver (Pacific Biosciences, CA, USA) with the base calls validated using Illumina MiSeq reads.

**Table 1:** Strain characteristics

Strain number	Accession number	Sero-type	Bovine abortion	Isolation year	Clade (Fig. 1)	Subspecies		1 % glycine tolerance	H_2_S production
						Genotype	Phenotype		
98/v445	LMBH00000000	B	No	1998	1	Cff	Cff	+	+
B0066	ERR419610	B	No	2013	1	Cff	Cff	+	+
B0130	ERR419638	B	No	2013	1	Cff	Cff	+	+
B0129	ERR419637	B	No	2013	1	Cff	Cff	+	+
S0478D	ERR419653	B	No	2011	1	Cff	Cff	+	+
B0042	ERR419595	B	No	2013	2	Cff	Cff	+	+
S0693A	ERR419284	B	No	2012	2	Cff	Cff	+	+
B0167	ERR460866	B	No	2013	2	Cff	Cff	+	+
B0168	ERR460867	B	No	2013	2	Cff	Cff	+	+
04/554	CP008808-CP008809	B	Yes	2004	2	Cff	Cff	+	+
B0047	ERR419600	B	No	2013	2	Cff	Cff	+	+
B0151	ERR419648	B	No	2013	2	Cff	Cff	+	+
B0152	ERR419649	B	No	2013	2	Cff	Cff	+	+
110800-21-2	LSZN00000000	A	No	2000	3	Cff	Cff	+	+
B0097	ERR419623	A	No	2013	3	Cff	Cff	+	+
BT 10/98	LRAL00000000	A	Unknown	1998	3	Cff	Cff	+	+
H1-UY	JYCP00000000	A	No	2013	4	Cff	n.a.	n.a.	n.a.
82-40	CP000487	A	No	1982	4	Cff	Cff	+	+
B0131	ERR419639	A	No	2013	4	Cff	Cff	+	+
Zaf 3	LREZ00000000	A	Yes	2006	5	Cfvi	Cfvi	−	+
CCUG 33872	LREU00000000	A	Unknown	1995	5	Cfvi	Cfvi	−	+
642-21	AJSG00000000	A	Unknown	n.a.	5	Cfvi	n.a.	n.a.	n.a.
ADRI 513	LRFA00000000	A	Unknown	n.a.	5	Cfv	Cfvi	−	+
CCUG 33900	LREV00000000	A	Yes	1995	5	Cfv	Cfv	−	−
LMG 6570	LREW00000000	A	Unknown	1953	5	Cfv	Cfv	−	−
B6	AJMC00000000	A	Unknown	n.a.	5	Cfv	n.a.	n.a.	n.a.
NCTC 10354	AFGH00000000	A	Unknown	1962	5	Cfv	Cfv	−	−
84-112	HG004426-HG004427	A	Unknown	1984	5	Cfv	Cfv	−	−
97/608	CP008810-CP008812	A	Yes	1997	5	Cfv	Cfv	−	−
B10	LRET00000000	A	Unknown	n.a.	5	Cfv	Cfv	−	−
WBT 011/09	LMBI00000000	A	Unknown	2009	5	Cfvi	Cfvi	−	+
Zaf 65	LREY00000000	A	Unknown	2007	5	Cfvi	Cff	+	+
97/532	LRER00000000	A	No	1997	5	Cfvi	Cfvi	−	+
01/165	CP014568-CP014570	A	No	2001	5	Cfvi	Cfvi	−	+
03/293	CP0006999-CP007002	A	Yes	2003	5	Cfvi	Cff	+	+
92/203	LRVL00000000	A	Yes	1992	5	Cfvi	Cfvi	−	+
03/596	LRAM00000000	A	Yes	2003	5	Cfvi	Cfvi	−	+
02/298	LRVK00000000	A	Yes	2002	5	Cfvi	Cfvi	−	+
ADRI 1362	LREX00000000	A	Unknown	1989	5	Cfvi	Cff	+	+
98/25	LRES00000000	A	Yes	1998	5	Cfvi	Cfv	−	−
INTA 99/541	ASTK00000000	A	Unknown	1999	5	Cfvi	n.a.	n.a.	n.a.
03-427	CP006833	A	No	2003	n.a.	Cft	Cft	+	+

n.a., not available; + positive; − negative; Cff, *C. fetus* subsp. *fetus*; Cfv, *C. fetus* subsp. *venerealis*; Cfvi, *C. fetus* subsp. *venerealis* biovar intermedius; Cft, *C. fetus* subsp. *testudinum*

The sequences with accession numbers starting with ERR are available from the European Nucleotide Archive (ENA) [18], and the remaining sequences are available from NCBI GenBank (Table 1). The following genomes were also included; Cff strain H1-UY (GenBank accession number JYCP00000000), Cfv strain 642–21 (GenBank accession number AJSG00000000), Cfv strain B6 (GenBank accession number AJMC00000000), Cfv strain NCTC 10354 (GenBank accession number AFGH00000000) and Cfvi strain 99/541 (GenBank accession number ASTK00000000).

The quality of the whole genome sequence data was assessed with the Checkm tool [19], showing a suitable completeness score of >96 % with 600 tested marker genes.

### Genome alignment and phylogenetic core genome SNP analysis

For phylogenetic core genome SNP analysis, whole genome sequences of 42 *C. fetus* isolates were aligned using Parsnp v1.2 [20]. We included Cft strain 03-427 as an outgroup for the phylogenetic core genome SNP analysis, but excluded this strain from visualization of the phylogenetic core genome SNP tree to get a better resolution of the Cff and Cfv branches. SNP discovery was focused on a comparison between Cff and Cfv strains; therefore, Cft strain 03-427 was replaced by Cff strain 82-40 as a reference for this analysis. Recombination regions in the core genome alignment were detected and visualized using Gubbins [21]. A phylogenetic tree was constructed using FastTree2 [22] with a generalized time-reversible model and gamma correction on the recombination-filtered SNPs in the core genome of all isolates, including Cft strain 03-427. The resulting tree was rooted on Cft strain 03-427 using Dendroscope [23] prior to visualization using iTOL [24].

### BEAST analysis

Recombination-filtered non-synonymous SNPs of the mammal-associated *C. fetus* isolates were extracted from the Gubbins results and used for divergence dating in BEAST [25], using the isolation dates as tip dates in the phylogenetic tree, as outlined in the BEAST manual with the following modifications: 10,000,000× sampling and a general time reversible (GTR) model plus gamma correction as the distance model. Combinations of a strict clock, log-normal clock, exponential clock and random local clock as the clock model and a constant population, exponentially-growing population, and a Bayesian skyline plot with six groups as demographic models were used. Maximum ESS values and lowest 95 % confidence intervals (CI) in the predicted divergence dates of the clades were obtained with a random local clock and a Bayesian skyline plot with four groups as the demographic model.

### Calculation of branch-specific dN/dS ratios

To calculate the dN/dS ratio per branch in the phylogenetic tree, we aligned the genomes of the mammal-associated *C. fetus* isolates with Parsnp v1.2 [20] and Cft strain 03-427 as outgroup. Synonymous and non-synonymous SNPs were determined on the basis of their location in the coding regions of the Cff strain 82–40 reference genome. Recombination regions were detected using Gubbins [21] and excluded from the alignment. Ancestral state reconstruction of the node sequences was performed using FastML [26] with a generalized time-reversible model and gamma correction. The dN/dS ratios were determined per branch between node sequences.

### Comparison of SNPs and genes with phenotypic characteristics of the strains

As traditional differentiation of *C. fetus* subspecies are based on the biochemical 1 % glycine tolerance and H_2_S production tests, the genomes were screened for genes and SNPs that were associated with these phenotypic characteristics, and tested for 1 % glycine tolerance and H_2_S production in cysteine-rich medium as described above. The protein-encoding gene presence and absence were determined using BLAST-based all vs all comparisons with Prokka-annotated genomes [22] using Roary [27], which clustered the proteins using MCL-edge [28]. A Fisher’s exact test was used to calculate the two-tail probability value (*p*) of respectively the detected SNPs and genes versus the outcome of the biochemical tests.

### Calculation of clinical association

A Fisher’s exact test was used to calculate the two-tail probability value (*p*) of SNPs and genes which were specifically found in strains that were isolated from bovine abortions. Significantly-associated SNPs or genes were checked for their presence in Cff strain 04/554, as this is a non-Cfv bovine clinical strain. SNPs and genes that were not present in this strain were excluded from this analysis, to separate clinical-associated from phylogenetically-associated SNPs and genes.

### Availability of data

Genome sequences are available from the European Nucleotide Archive (ENA) and from NCBI GenBank, with the accession numbers listed in Table 1.

## Results

### Phylogenetic analysis of the core genome SNPs

**Fig. 1:**
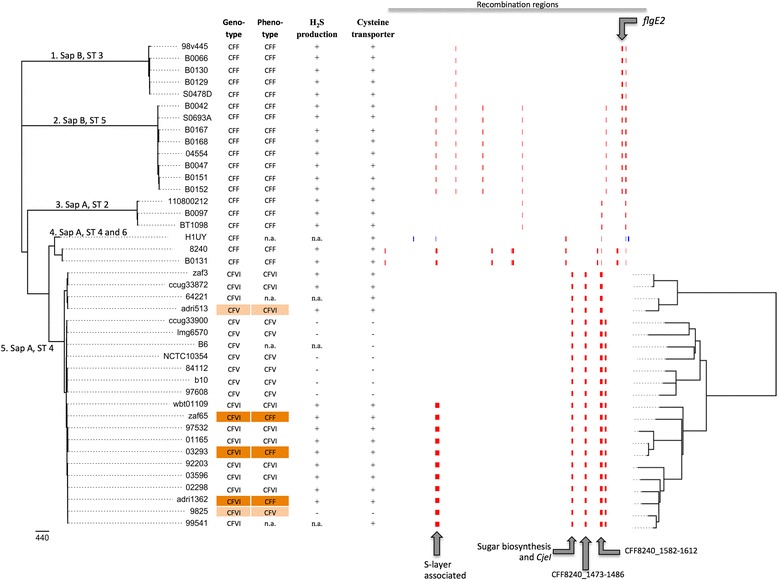
ML tree of *C. fetus* core genome and mammalian associated *C. fetus* recombination regions. Cft 03-427 was excluded from the visualization. Tree branches are labeled with the clade numbers, serotype and MLST STs of the *C. fetus* strains. Subspecies identification (genotype and phenotype), H_2_S production test results (+ positive, − negative), the presence of the putative cysteine transporter (+ present, − absent) and the Gubbins predicted recombination regions in the mammalian associated *C. fetus* core genome are shown, with on the right side a zoomed ML tree of clade 5. Gene *flgE2* encodes a flagellar hook protein and *cjeI* a type II R-M system protein. Scale bar at the bottom left represents the nucleotide substitutions per site

Phylogenetic analysis, based on core genome SNPs using Cft as the outgroup, showed that the mammal-associated Cff and Cfv genomes group into five distinct clades (Fig. 1). The division of clades was consistent with the serotypes of the strains; clades 1 and 2 consist of serotype B strains and clades 3, 4 and 5 consist of serotype A strains. The division of clades was also consistent with the classification of MLST sequence types (STs), except for Cff strain H1-UY, which has the Cfv-associated MLST ST-4 genotype [13]. Interestingly, SNP phylogeny showed the divergence of clade 4 and clade 5 from a common Cff ancestor. Clade 5 consists of phenotypically-identified Cff, Cfvi and Cfv strains (Table 1) without a clear separation in the phylogeny. However, when the genotypic characterization is used [8, 16], clade 5 consists exclusively of Cfv and Cfvi strains and clade 1–4 of Cff strains.

### BEAST analysis determined that diversification of the mammal-associated C. fetus is a relatively recent event under disruptive selection pressure

**Fig. 2:**
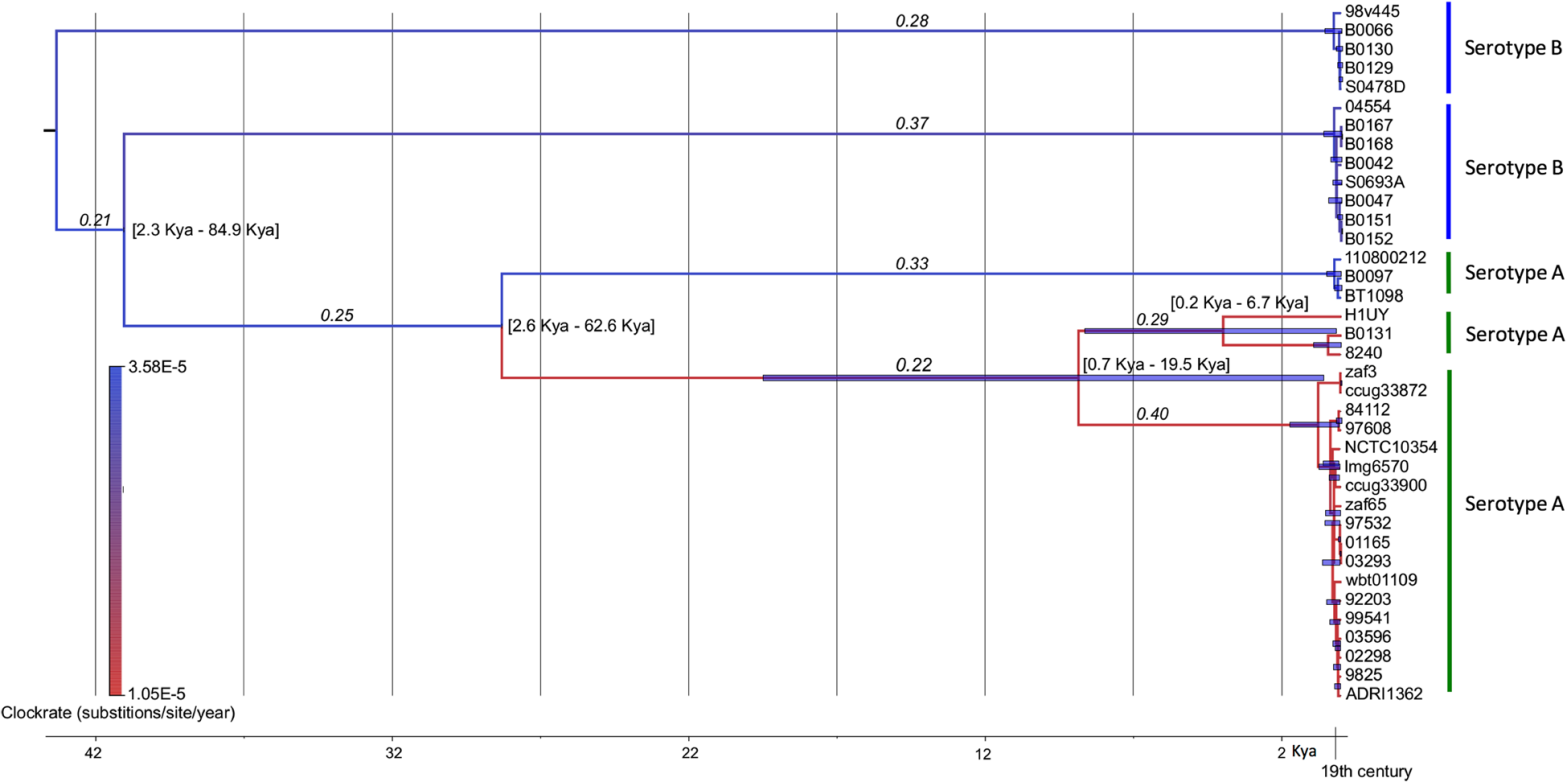
BEAST tree with divergence dates. BEAST analysis 95 % CI divergence dates are shown within brackets and with blue bars on branch nodes. dN/dS ratios are shown in italics on the branches. Branches are colored according the clock rates, represented as substitutions per site per year. Scale bar at the bottom represents thousand years ago (Kya)

We used BEAST to determine the divergence date of the different clades (Fig. 2). The topology of the BEAST tree was slightly different than the maximum likelihood (ML) SNPs tree (Fig. 1), especially for the serotype B strains. This may be either the result of using only synonymous SNPs in the BEAST analysis or because of the differences between ML methods and methods based on coalescence. The 95 % confidence interval (CI) of the BEAST analysis was too large to estimate the divergence date of clade 2 and clades 3, 4 and 5 [95 % CI 2.3 Kya – 84.9 Kya] and the divergence date of clades 4 and 5 [95 % CI 0.7 Kya – 19.5 Kya] (Fig. 2). Interestingly, the BEAST analysis showed that all currently circulating strains in clades 1, 2, 3 and 5 diverged very recently from each other.

Cfv is restricted to the bovine genital tract, but Cff can be isolated from different animal species, e.g. cattle, sheep, goats, pigs, horses, and humans [2]. Therefore, we attempted to investigate whether this change in niche preference has resulted in increased evolutionary pressure on Cfv. We estimated the rate of molecular evolution by comparing the ratio of non-synonymous (dN) versus synonymous (dS) SNPs (Fig. 2). The dN/dS ratio was the highest in the strains of clade 5 (0.40) compared to the ratio of the strains of clade 4 (0.29) and in all branches preceding, based on node sequences reconstructed using FastML (Fig. 2). An increased dN/dS ratio (>0.2) is indicative for recently diversifying genomes, whereas lower dN/dS ratios (0.04–0.20) correspond to older SNPs and more diverged genomes [29]. All clades have a dN/dS ratio > 0.2, with the highest dN/dS ratio of clade 5 of 0.40, showing that the strains of clade 5 are evolving under relatively more diversifying selection than the strains of the other clades. The estimated molecular clock rate for the mammal-associated *C. fetus* strains was 1.5–3.5 × 10^−2^ substitutions per kb per year, which corresponds with the estimated molecular clock rate of *C. jejuni* of 1.86–5.81 × 10^−2^ per kb/year [30].

### Clade-specific SNPs and orthologs

The numbers of SNPs and orthologous genes (orthologs) specifically present in each clade (Fig. 1) are presented in Table 2, as well as the specific SNPs and orthologs for clades representing the serotypes and subspecies of the strains. The details and annotation of the detected SNPs and orthologs are listed in Additional file 1: Table S1.

**Table 2:** Core genome SNPs specific for *C. fetus* clades

Clade	Representing	Number of clade-specific SNPs	Number of clade-specific orthologs (annotation)
1	Cff serotype B	1547	2 (hypothetical)
2	Cff serotype B	1703	41 (fic, transposase, hypothetical)
3	Cff serotype A	1430	6 (R-M system type I and III)
4	Cff serotype A	121	0
5	Cfv and Cfvi	282	0
5	Cfv	5	6
1–2	Cff serotype B, total	571	14 (toxin/antitoxin, R-M, S-layer associated)
3–4	Cff serotype A, total	83	14 (glycosyltransferase, methyltransferase, ketoreductase)

Cff, *C. fetus* subsp. *fetus*; Cfv, *C. fetus* subsp. *venerealis*; Cfvi, *C. fetus* subsp. *venerealis* biovar intermedius

Clades 1–2 consisted only of Cff serotype B strains. For clade 1, 1547 SNPs and 2 orthologs were found, specifically present in clade 1 strains (Table 2, Additional file 1: Table S1). The two clade 1-specific orthologs were hypothetical proteins with unknown function. Specific for clade 2, 1703 SNPs and 41 orthologs were found (Table 2, Additional file 1: Table S1). The specific orthologs were located in a region encoding mainly hypothetical proteins, as well as a Fic domain protein and a transposase. This clade 2-specific region is chromosomally located in Cff strain 04/554 (CFF04554_0637 - CFF04554_0684). All strains of both clades 1 and 2 (Cff serotype B) contain 571 specific SNPs and 14 specific orthologs. The clade 1 and 2-specific orthologs included a toxin/antitoxin system (CFF04554_0478 and CFF04554_0479), restriction-modification (R-M) system-associated genes (CFF04554_1799, CFF04554_0318 and CFF04554_0319) and S-layer associated genes (CFF04554_1746, CFF04554_0481 and CFF04554_0484).

Clades 3 and 4 consist of Cff serotype A strains. Clade 3 contains 1430 specific SNPs and six specific orthologs (Table 2, Additional file 1: Table S1). The specific orthologs encoded hypothetical proteins of unknown function, as well as R-M type I (CFF8240_0988) and R-M type III genes. For clade 4, 121 specific SNPs and no specific orthologs are found. All strains of clades 3–4 (Cff serotype A) contain 83 SNPs and 14 specific orthologs that are mainly located in one region (CFF8240_1591 – CFF8240_1608) that includes a glycosyltransferase, a methyltransferase and a ketoreductase.

Clade 5 consists of both genotypically-identified Cfv and Cfvi strains. For this clade, 282 specific SNPs were found (Additional file 1: Table S1). The genotypically-identified Cfv strains of clade 5 contain five specific SNPs and six specific orthologs. The specific orthologs encoded hypothetical proteins, a transcriptional regulator (CFV97608_1300), a resolvase and a transposase.

### Analysis of SNP regions that are subject to recombination

Interestingly, 42 % (119/282) of the SNPs differing between clades 1–4 and clade 5 are located in regions <1 kb apart. This is suggestive of recombination, since on average a SNP would be expected to occur approx. every 6.5 kb, based on the number of SNPs identified here in genomes of approx. 1.85 mbp. (1.85 mbp./282 SNPs = 6.5 kb/SNP). We predicted potential recombination regions in the mammal-associated *C. fetus* core genome alignment with Gubbins [21] and visualized the results (Fig. 1). Using this analysis, one region showed recombinations in all strains of clades 1–4 and three recombination regions were found in all strains of clade 5.

The recombination region common to all strains of clades 1–4 encoded the flagellar hook gene *flgE2* (CFF8240_1769) (Fig. 1). All strains of clade 5 contained a recombination region (CFF8240_1393-CFF8240_1398) with a type II R-M gene (*cjeI*) (CFF8240_1393) and genes involved in sugar biosynthesis, including: a NAD-dependent epimerase/dehydratase (CFF8240_1396); a nucleotide sugar dehydrogenase (CFF8240_1397); and a polysaccharide biosynthesis protein (CFF8240_1398). Another clade 5-specific recombination region is CFF8240_1473-CFF8240_1486 that encodes: leader peptidase A (LepA; CFF8240_1473); ribose-phosphate pyrophosphokinase (Prs; CFF8240_1474); a subunit of aspartate carbamoyltransferase (PyrB; CFF8240_1475), a formate dehydrogenase subunit (FdhC; CFF8240_1482) and a glutamate synthase subunit (GltD; CFF8240_1486). In all strains of clade 5, a recombination region was found (CFF8240_1582-CFF8240_1612), encoding: several radical SAM domain proteins; two transketolase subunits (CFF8240_1587 and CFF8240_1589); a methyltransferase (CFF8240_1590); and two glycosyltransferases (CFF8240_1607 and CFF8240_1612). Additionally, the S-layer (*sap)* region (CFF8240_0455-CFF8240_0501) was also identified by Gubbins as a recombination region, although the *sap* locus itself is not included in the core genome alignment due to assembly issues of this region [12].

### Deletion of a putative cysteine transporter is associated with H_2_S production-negative strains

Currently, the OIE-prescribed biochemical tests to differentiate Cff, Cfv and Cfvi consist of determining tolerance to 1 % glycine and production of hydrogen sulfide (H_2_S) from L-cysteine [7]. We analyzed the genomes of the *C. fetus* strains for the presence of specific genes associated with these phenotypic characteristics. Interestingly, H_2_S negative strains (Table 1) did not encode two subunits of an amino-acid ABC transporter (CFF8240_0780 and CFF8240_0781), that together with an ATP-binding subunit (CFF8240_0779) putatively form an amino-acid ABC transport system involved in cysteine transport (Kegg module cff_M00234) (Additional file 2). The absence of the two ABC transporter genes in the *C. fetus* H_2_S negative strains possibly explains the phenotypic characteristic of these strains. Cfvi strain 98/25 was described before as H_2_S positive [8, 9]; however, in this study, the isolates that were sequenced were biochemically characterized and the sequenced isolate of strain 98/25 was H_2_S production negative, in accordance with the absence of the putative cysteine transporter in this genome.

### Conjugative transfer region associated with strains from bovine abortions

The *C. fetus* strains were arranged according to their clinical features: nine strains were from bovine abortions; seventeen strains were from screenings; and for fifteen strains, the clinical features were unknown (Table 1). No SNPs were found that were both present in the only known Cff strain from a bovine abortion (strain 04/554) and significantly present in the Cfv/Cfvi strains from bovine abortions. However, one region was significantly present in all strains from bovine abortions (*p* < 0.05); this region contains genes encoding a filamentation induced by cyclic AMP (Fic) domain protein (CFV97608_b0017), a DNA-binding protein (CFV97608_b0010) and conjugative transfer (*tra*) proteins (CFV97608_b00014, CFV97608_b0015, CFV97608_b0021). This region is located in Cff strain 04/554, Cfv strain 97/608 and Cfv strain 84-112 on a megaplasmid or an extra-chromosomal element [17]. This *tra/trb* region is present in 11 *C. fetus* strains isolated from non-abortion cases; Cff strain 98/v445 and Cfv/Cfvi strains CCUG 33872, 642-21, ADRI 513, B10, 84-112, LMG 6570, NCTC 10354, Zaf 65, ADRI 1362 and 99-541 (Additional file 1).

## Discussion

The core genome SNP analysis of the mammal-associated *C. fetus* strains identified a large number of SNPs. Since available PCR methods and MLST typing schemes are not able to identify *C. fetus* strains correctly to subspecies level [13, 15], Cfv/Cfvi-specific SNPs could be used to develop new diagnostic methods, such as a SNP probe-PCR, or to improve the current MLST scheme. However, two drawbacks of a PCR based on Cfv/Cfvi-specific SNPs are that the stability of such SNPs is unknown and that the design of a reliable PCR based on specific SNPs requires a high degree of optimization [31, 32]. In a previous study, we presented the division of the mammal-associated *C. fetus* strains into two different clusters, based on a core gene alignment of five Cff and 18 Cfv/Cfvi strains [9]. In this study, 19 Cff strains and 22 Cfv/Cfvi were included*,* which gave a better resolution of the branches with Cff strains, when compared to a previous analysis with five Cff strains [9]. The current core genome SNP analysis separated the *C. fetus* strains into five different clades, that contain either the genotypically-identified Cff (four clades) or Cfv/Cfvi (one clade) strains. We demonstrated that the biochemical differentiation of the Cff, Cfv and Cfvi strains is not supported by the core genome SNP phylogeny: clade 5 consists of phenotypically-identified Cff, Cfv and Cfvi strains without a congruent separation in the phylogeny of these strains This showed that the phenotypic separation of the mammal-associated *C. fetus* strains is not supported by the core genome SNP phylogeny, which gives rise to the consideration if the current phylogenetic subspecies differentiation is still reasonable, as previously mentioned [9].

### Forces of Cff and Cfv diversification

Phylogenetic analysis indicated that Cfv/Cfvi clade 5 and Cff clade 4 have a common Cff ancestor. No association of the clades with geographic origin and/or host specificity was observed. Interestingly, the short terminal branches and recent diversification suggest that the isolates within each clade, except for strain H1-UY, have diverged recently. This is in contrast to what is observed in Cft, where terminal branches of the isolates are much longer and a much higher genome diversity is observed [11]. The world-wide spread of the mammal-associated *C. fetus* strains is potentially associated with the improvements in cattle breeding by selection and cross-breeding which started around the 1700s and 1800s, followed by extensive spread of high-producing dairy cows and beef cattle [33], which may have carried only a very limited number of *C. fetus* clones. We suggest that this spread from a limited number of sources has resulted in our current observation of the highly clonal nature of mammal-associated *C. fetus*. Alternatively, the observation of only recently diverged strains is a result of selective sweeps, when a population member with an advantageous trait will take over the population before the trait can spread to other members [34]. It is unlikely that selective sweeps occurred in the mammal-associated *C. fetus* population, since cattle are intensively monitored worldwide for the presence of *C. fetus* subsp. *venerealis* and only one non-clonal *C. fetus* strain was found (H1-UY). Cff strain H1-UY was isolated from the blood of a rural worker in 2013, who was diagnosed with cellulitis and was in daily contact with cattle [13].

The rates of changes at non-synonymous and synonymous SNPs indicate whether a gene is under purifying or diversifying selection [35, 36] and is expressed in the dN/dS ratio. The dN/dS ratio of bacterial genes under stabilizing selection falls generally within the range 0.04–0.2 [37]. In the mammal-associated *C. fetus*, the dN/dS ratios were all > 0.2, showing that all genomes are under diversifying selection. The highest dN/dS ratio was found for the clade 5 strains (0.40) (Fig. 2). This high ratio may be due to the genetic features of these strains: they have no CRISPR-cas systems and the clade 5-strains have 24 specific SNPs in the type II R-M system gene *cjeI* compared to the other strains (Additional file 1: Table S1). Type II R-M systems can undergo major changes in specificity by recombination events as shown for *Helicobacter pylori* [38] and R-M type II systems may play a role in plasmid transformation, as described for *C. jejuni* where knockout mutagenesis of gene *cjeI* resulted in a strain with a 1,000-fold-enhanced transformation efficiency [39]. It is unknown whether these SNPs influence the functionality or specificity of this type II R-M system in *C. fetus* strains, but the clade 5-strains are possibly more susceptible to insertion of foreign DNA, since the genomes contain genomic islands (GIs) encoding T4SSs, phages and insertion sequences [9, 17].

### H_2_S negative phenotype of Cfv strains associated with loss of a putative cysteine transporter

The original classification of the mammal-associated *C. fetus* subspecies Cff and Cfv are based on the 1 % glycine tolerance test [40]. Cfv strains can be discriminated from Cfvi strains with the H_2_S production test [5]. We show that the H_2_S negative *C. fetus* strains have lost a putative cysteine transporter. Without the encoded transporter, the cells are possibly less capable of importing cysteine, which under normal situations is reduced while forming H_2_S. The H_2_S-negative Cfv strains have a niche restriction to the genital tract of cattle, whereas the H_2_S-positive Cfvi and Cff strains are not restricted to the genital tract and assumed to be able to colonize the intestines as well [5]. One may speculate that the Cfv strains have a defect causing the incapability to grow outside of the genital tract, but it is unknown if this partial deletion of the putative cysteine transporter can be associated with the niche restriction of these strains.

### Virulence-associated genes specific for clades and strains from bovine abortions

Since the first description of the *C. fetus* subspecies in 1959, it is presumed that Cfv cause disease in the genital tract of cows, like enzootic venereal sterility and abortions in pregnant cows, and that Cff only cause sporadic abortions [40]. It is unknown which genomic features are responsible for the pathogenicity of the *C. fetus* strains, but potential candidates are the surface layer of the *C. fetus* cells [10, 41] and type IV secretion systems (T4SSs) [17, 42, 43]. We studied the genomes of nine strains from bovine abortions and observed that, in addition to the S-layer proteins and T4SSs, all the strains from bovine abortions contain a region encoding conjugative transfer (*tra*) proteins. This *tra* region is located in the closed genomes of strains 04/554, 97/608 and 84-112 on a plasmid/ICE, which also contain a *trb*-T4SS and *fic* genes. The fic domain proteins encoded by this region are of potential interest, because fic domain proteins have an immune-modulatory function by influencing the cytoskeleton organization of the host cells [44] and are translocated by T4SSs in *Legionella pneumophila*, *Coxiella burnetii* and *Bartonella henselae* [43], which has also been proposed for *C. fetus* strains [45]. The *trb/tra* genes are also present in 11 *C. fetus* strains isolated from non-abortion cases (Additional file 1). It is possible that these strains can also cause bovine abortions; however, more studies, specifically animal experiments investigating the virulence of *C. fetus* strains are required to assess this hypothesis.

## Conclusion

Phylogenetic core genome SNP analysis divided the mammal-associated *C. fetus* strains into five different clades, which were consistent with the serotypes, but not with the phenotypes of the strains. BEAST analysis showed that the clade with genotypically-identified Cfv/Cfvi strains has evolved from a Cff ancestor under diversifying selection. Phylogenetic analysis of the core genome SNPs did not differentiate H_2_S-negative Cfv from H_2_S positive Cfvi strains. The partial deletion of a putative cysteine transporter is observed in all H_2_S negative Cfv strains.

## Additional files

Additional file 1: Table S1. Table with details of *C. fetus* SNPs and genes, specific for clades and bovine abortion strains. (XLSX 4167 kb) Additional file 2: Figure S1. Schematic representation of the putative cysteine transporter genes CFF8240_0779 - CFF8240_0781 (white) in strain Cff 8240 with flanking genes *rarD* and *psgA* (grey). The start and stop positions of respectively *rarD* and *psgA* are shown in italics. Cfv strain 97/608 lacks one complete ORF and contain a remnant of one ORF of the putative cysteine transporter. Shown are the similar deletion sites sequences in the Cfv strains containing the incomplete transporter. (PDF 149 kb)

## Abbreviations

BGC: Bovine Genital Campylobacteriosis
*C. fetus*: *Campylobacter fetus*
Cff: *C. fetus fetus*
Cft: *C. fetus testudinum*
Cfv: *C. fetus venerealis*
Cfvi: *C. fetus venerealis* biovar intermedius
GI: Genomic island
Kya: Thousand (Kilo) years ago
SNP: Single nucleotide polymorphism
T4SS: Type IV secretion system
