# Supplementary material for: Whole genome sequence analysis indicates recent diversification of mammal-associated Campylobacter fetus and implicates a genetic factor associated with H2S production
Source: BMC Genomics. 2016 Sep 6;17(1):713. doi: 10.1186/s12864-016-3058-7 (PMC5013579; doi:10.1186/s12864-016-3058-7)
Supplement: Additional file 2: Figure S1. — Schematic representation of the putative cysteine transporter genes CFF8240_0779 - CFF8240_0781 (white) in strain Cff 8240 with flanking genes rarD and psgA (grey). The start and stop positions of respectively rarD and psgA are shown in italics. Cfv strain 97/608 lacks one complete ORF and contain a remnant of one ORF of the putative cysteine transporter. Shown are the similar deletion sites sequences in the Cfv strains containing the incomplete transporter. (PDF 149 kb) [file 12864_2016_3058_MOESM2_ESM.pdf]

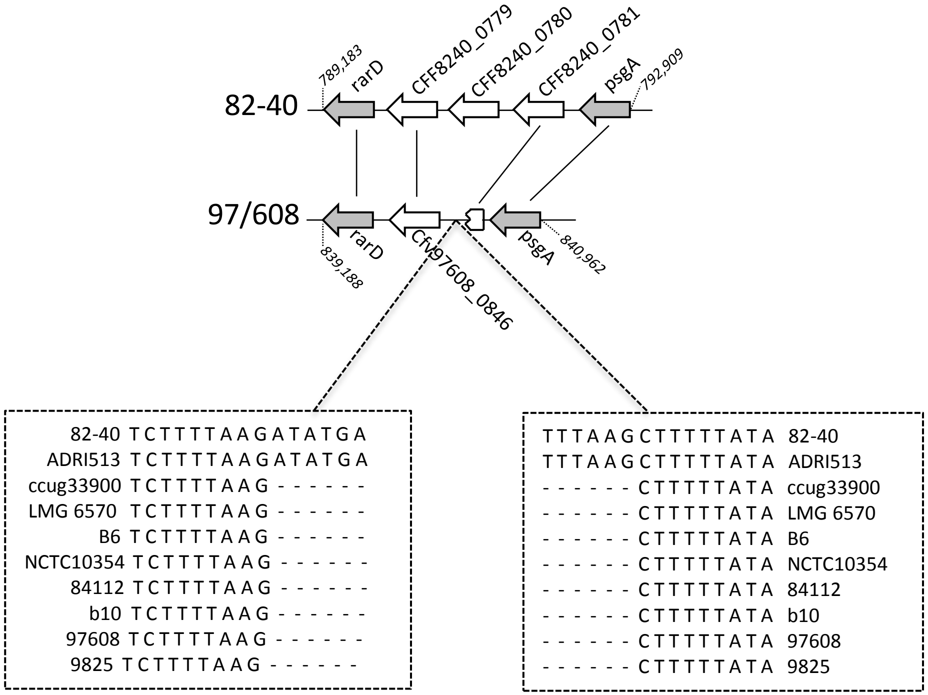
82-40
789,183
rarD
CFF8240_0779
CFF8240_0780
CFF8240_0781
psgA
792,909
97/608
rarD
839,188
Cfv97608_0846
psgA
840,962
82-40 TCTTTTAAGATATGA
ADRI513 TCTTTTAAGATATGA
ccug33900 TCTTTTAAG------
LMG 6570 TCTTTTAAG------
B6 TCTTTTAAG------
NCTC10354 TCTTTTAAG------
84112 TCTTTTAAG------
b10 TCTTTTAAG------
97608 TCTTTTAAG------
9825 TCTTTTAAG------
TTTAAGCTTTTTATA 82-40
TTTAAGCTTTTTATA ADRI513
------CTTTTTATA ccug33900
------CTTTTTATA LMG 6570
------CTTTTTATA B6
------CTTTTTATA NCTC10354
------CTTTTTATA 84112
------CTTTTTATA b10
------CTTTTTATA 97608
------CTTTTTATA 9825
